# Supplementary figures and images for: Dynamic brain connectivity predicts emotional arousal during naturalistic movie-watching
Source: PLoS Comput Biol. 2025 Apr 11;21(4):e1012994. doi: 10.1371/journal.pcbi.1012994 (PMC12058195; doi:10.1371/journal.pcbi.1012994)

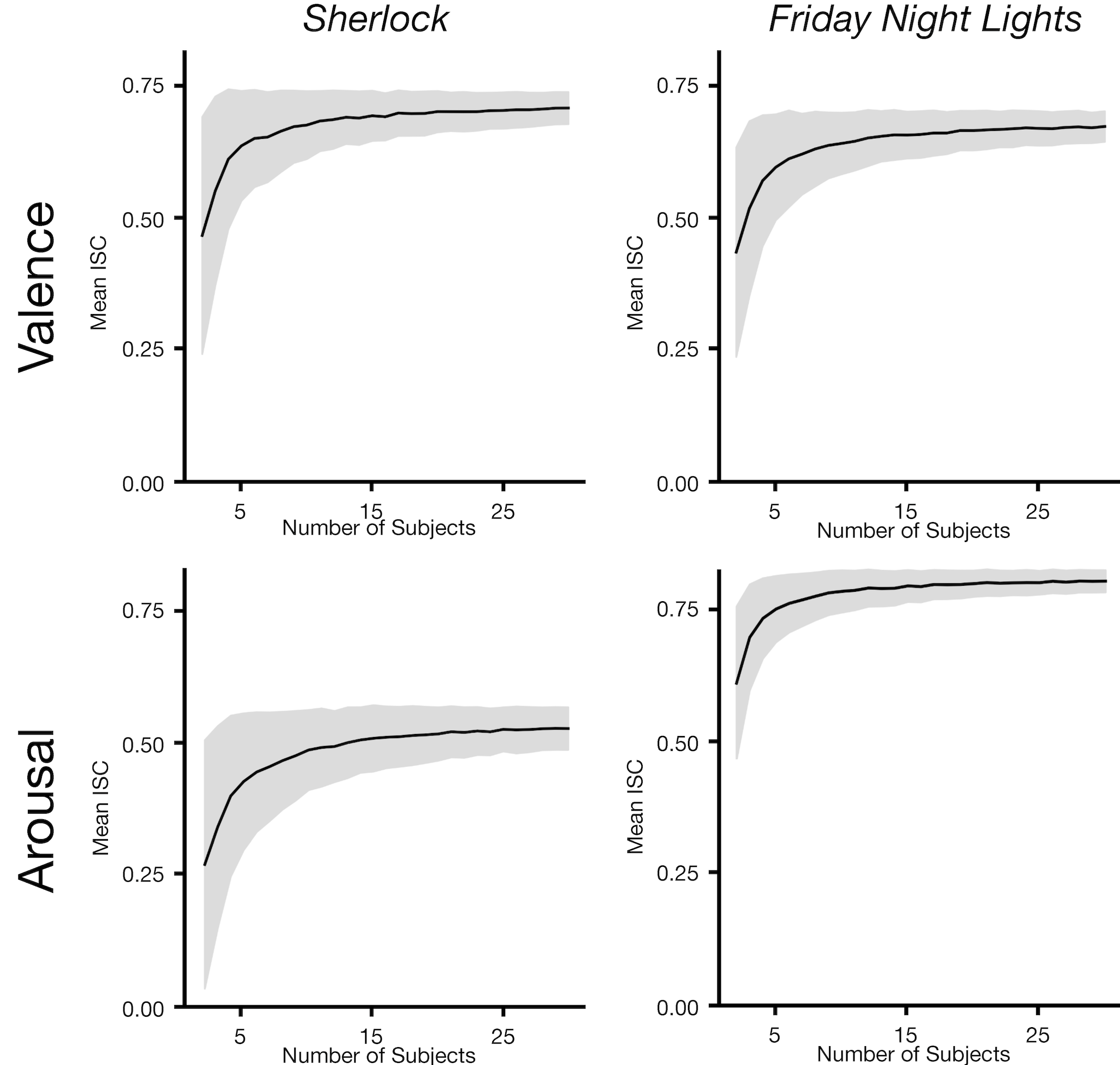

Supplement: S1 Fig — The x-axis represents the number of subjects. In each condition, the number of subjects, k, increased from 2 to 30. Corresponding to the point k on the x-axis, k subjects were randomly selected from all subjects for 1000 times with replacement, where each time the Fisher’s z-transformed group-average ISC was computed. The gray area represents the standard deviation of the distribution of permutations. The group-average rating similarity stabilizes as the number of subjects increases, suggesting it is unlikely for the group-average behavioral rating to increase in precision with more subjects. (TIFF) [file pcbi.1012994.s001.tiff]

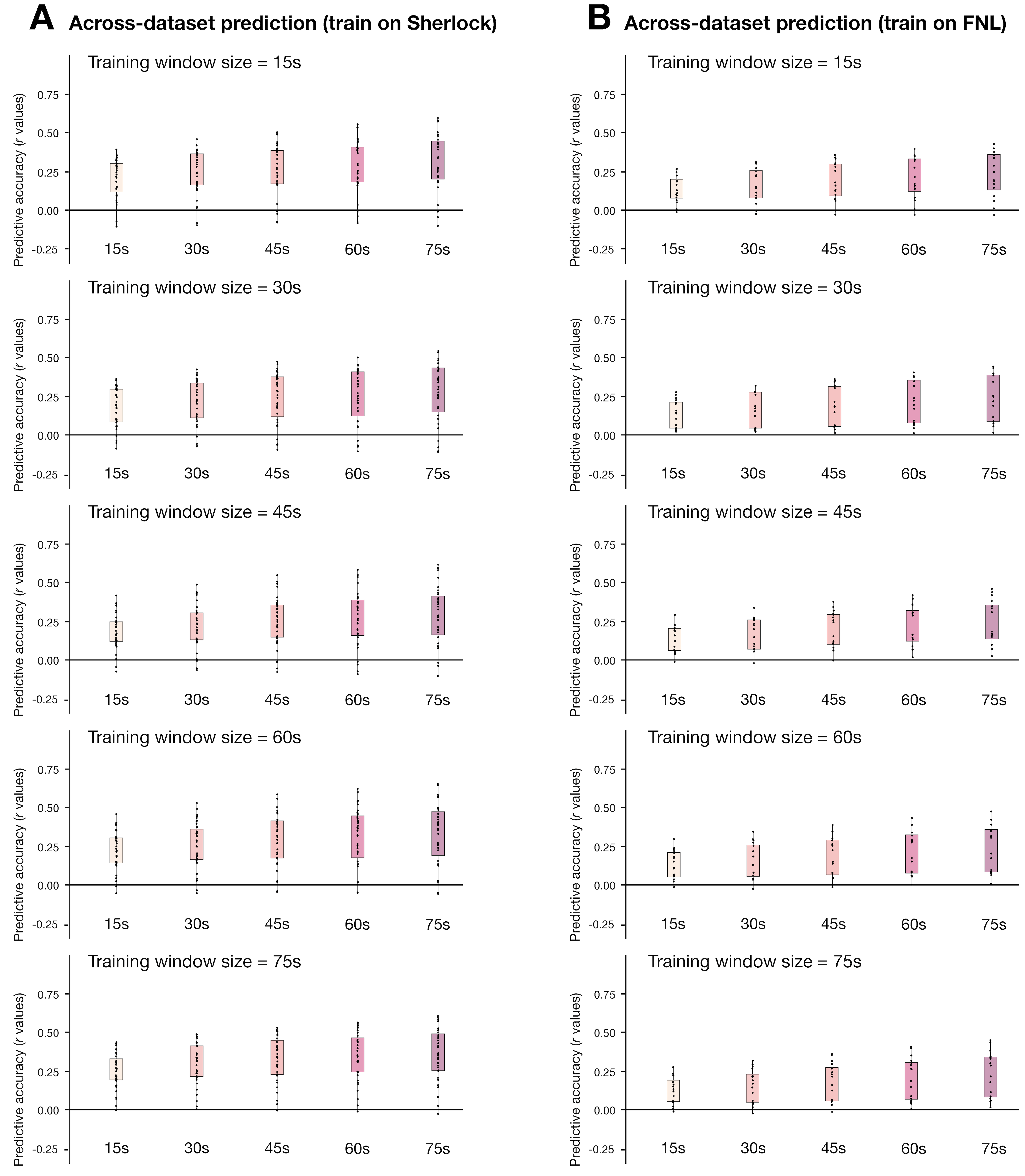

Supplement: S2 Fig — The x-axis represents the number of subjects. In each condition, the number of subjects, k, increased from 2 to 30. Corresponding to the point k on the x-axis, k subjects were randomly selected from all subjects for 1000 times with replacement, where each time the Fisher’s z-transformed group-average ISC was computed. The gray area represents the standard deviation of the distribution of permutations. The group-average rating similarity stabilizes as the number of subjects increases, suggesting it is unlikely for the group-average behavioral rating to increase in precision with more subjects. (TIFF) [file pcbi.1012994.s002.tiff]

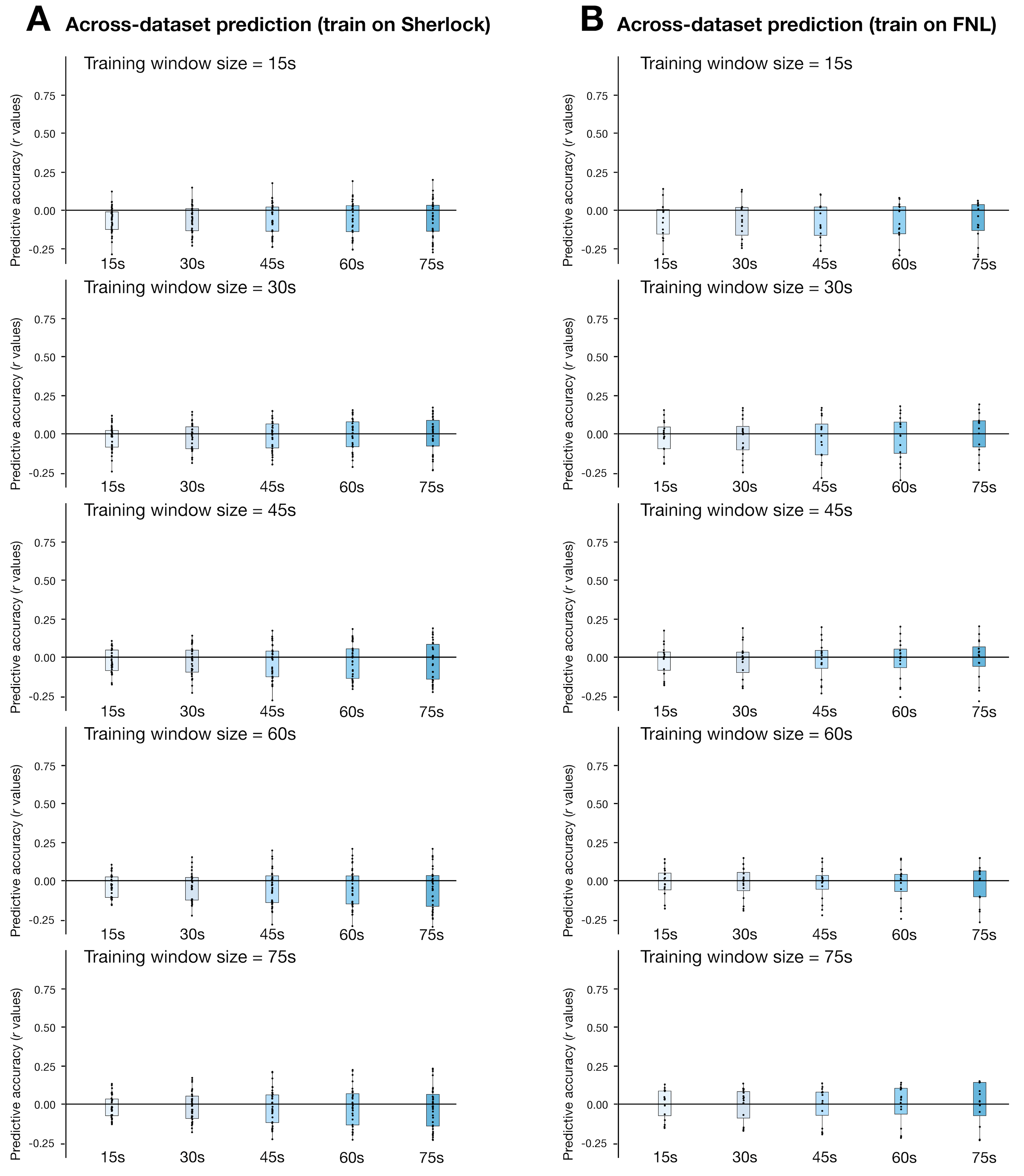

Supplement: S3 Fig — Model predictive accuracy when training on Sherlock and testing on Friday Night Lights (A) and when training on Friday Night Lights and testing on Sherlock (B). We extracted dynamic functional connectivity patterns from both Sherlock and Friday Night Lights using 5 different sizes of tapered sliding window: 15s, 30s, 45s (the original size in the main analysis), 60s and 75s, and tested each model on test data at each window size, resulting in 50 conditions (2 datasets x 5 window sizes at training x 5 window sizes at testing). None of the 50 conditions showed above chance predictive accuracy in predicting valence. (TIFF) [file pcbi.1012994.s003.tiff]

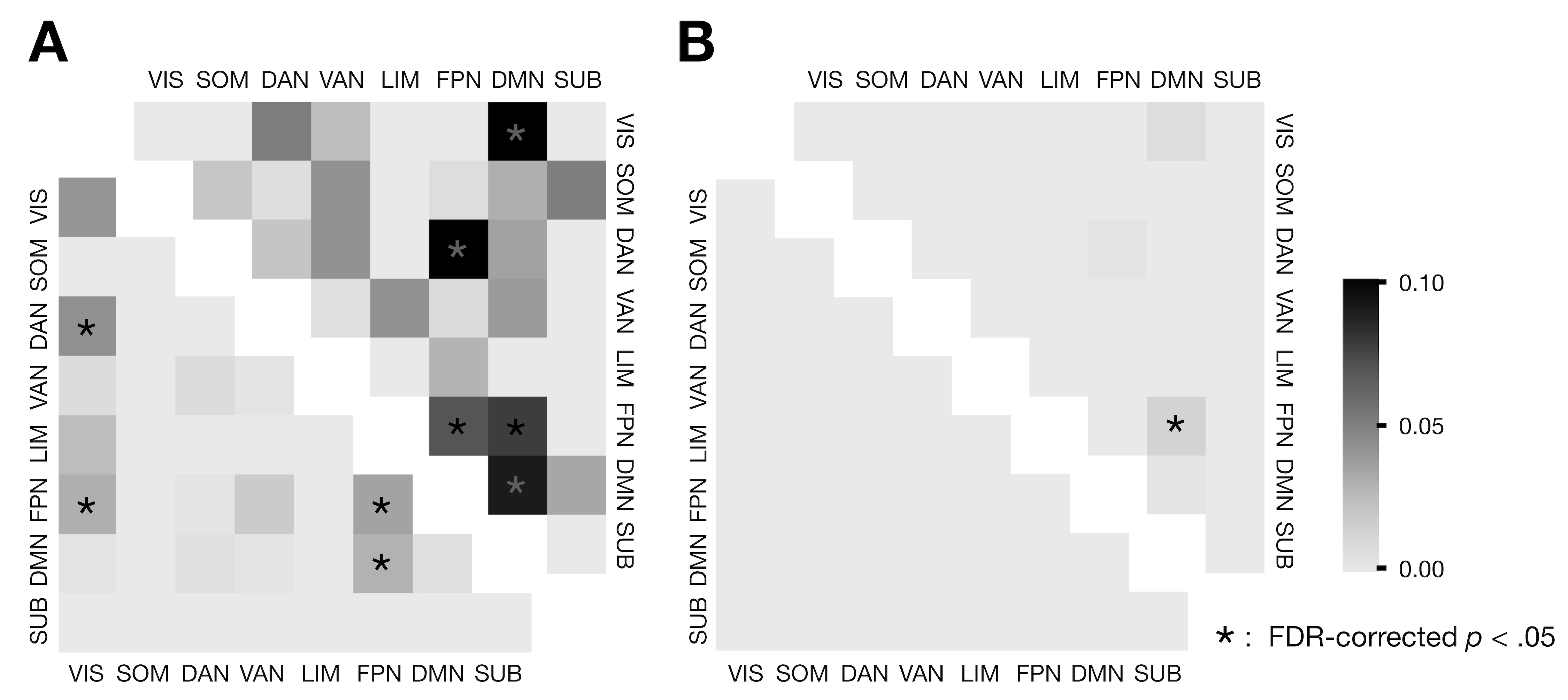

Supplement: S4 Fig — Engagement network acquired from Song et al., 2021 [36]. In each figure, the upper triangle represents the network which contains functional connections that positively correlate with both arousal and engagement. The lower triangle represents the network which contains functional connections that negatively correlate with both arousal and engagement. Each cell represents the proportion of selected FCs among all possible FCs between each pair of functional networks. Networks pairs with above-chance selected FCs are indicated with an asterisk (one-tailed t-test, fdr-corrected p < .05). A. The overlap between the arousal network and engagement network in the Sherlock dataset. The connections between DMN and FPN, between DMN and VIS, between DAN and FPN as well as connections within the DMN and FPN positively predicted both arousal and engagement. The connections between VIS and DAN, between VIS and FPN, between FPN and DMN, as well as connections within the FPN negatively predicted both arousal and engagement (FDR-corrected P < .05). B. The overlap between the Sherlock-Friday Night Lights arousal network and Sherlock-Paranoia engagement network. Only the connection between DMN and FPN positively predicted both arousal and engagement in this cross-dataset manner. (TIFF) [file pcbi.1012994.s004.tiff]

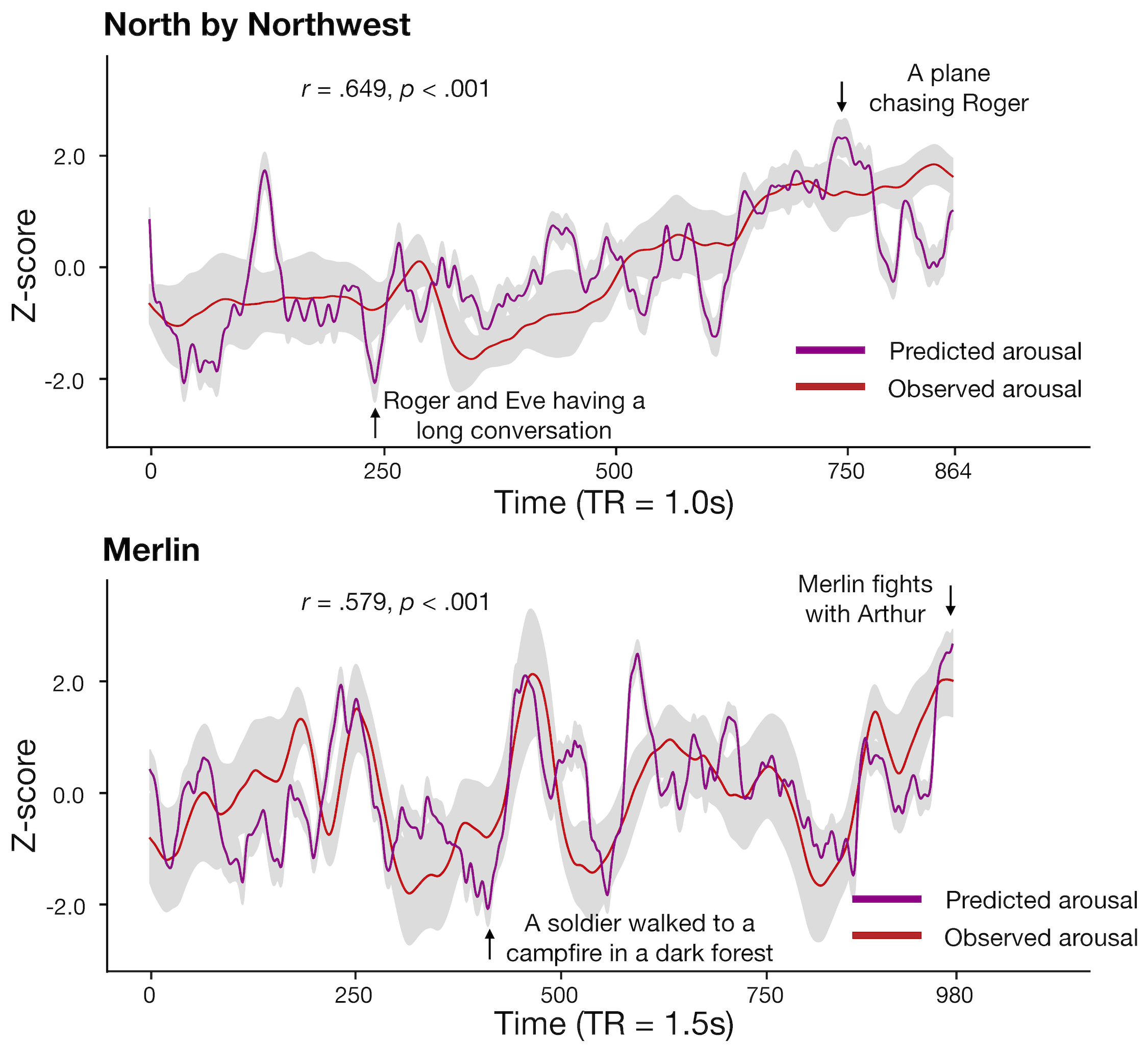

Supplement: S5 Fig — Model predicted time courses across participants watching the same movie was averaged. The arousal time course for both North by Northwest and Merlin was predicted from a model trained on the overlap arousal network from Sherlock and Friday Night Lights. This averaged predicted arousal time course (predicted arousal) significantly correlated with the group-average arousal rating from a separate group of individuals (observed arousal). Model-predicted arousal time courses also corresponded with the plot of each movie. The gray bands indicate the standard deviation of predicted arousal across participants at each time point. In North by Northwest, the most arousing moment predicted by the model occurred in the scene when the protagonist was being chased by a plane, while the least arousing moment occurred during a long conversation between characters; In Merlin, the most arousing moment predicted by the model occurred when the two main protagonists had a brawl in a tavern, while the least arousing moment occurred when a nameless soldier walked towards a campfire. Images in the figures are blurred for copyright reasons; in the experiment, movies were shown at high resolution. (TIFF) [file pcbi.1012994.s005.tiff]

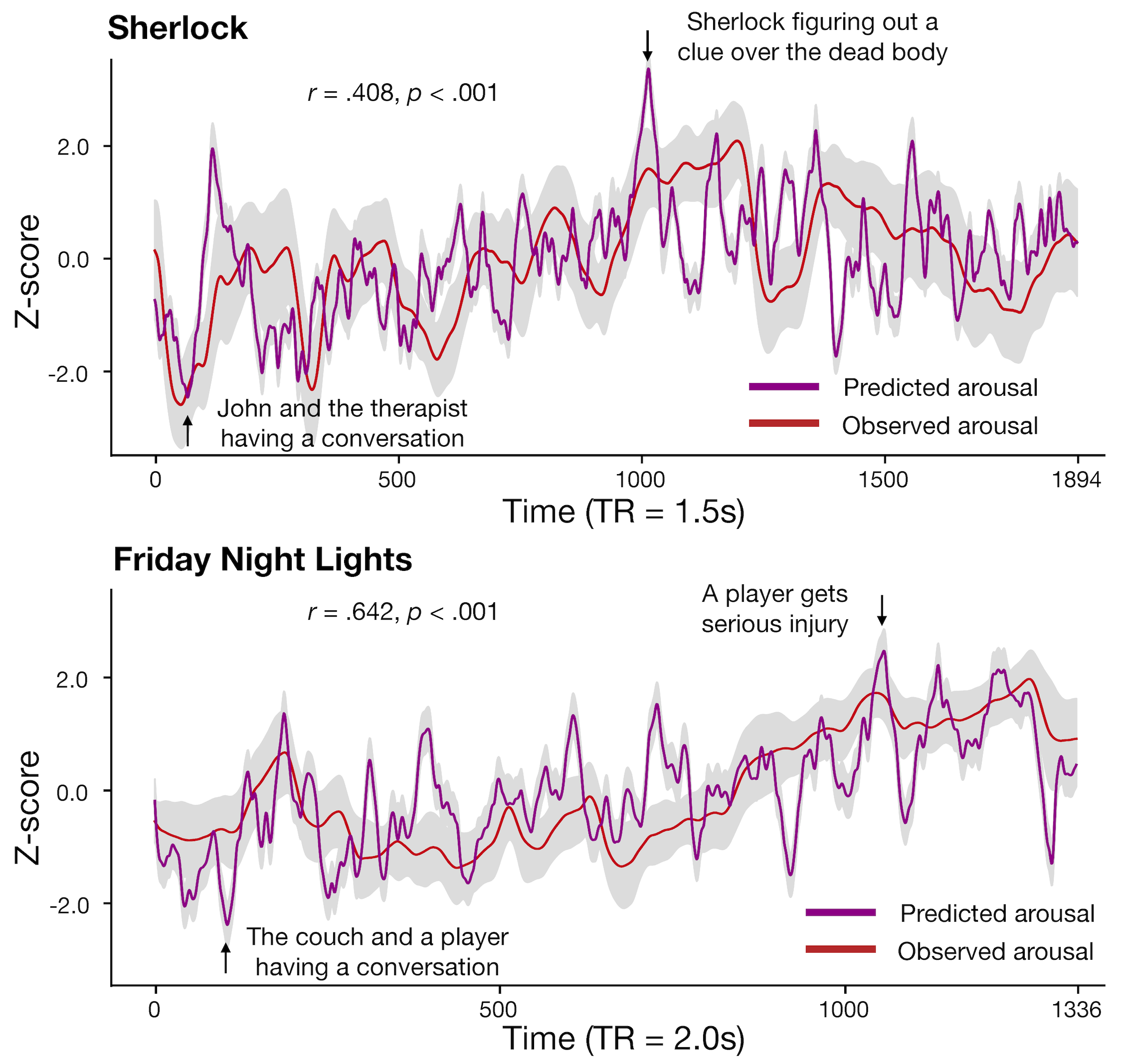

Supplement: S6 Fig — Model predicted time courses across participants watching the same movie was averaged. The arousal time course for Sherlock was predicted from a model trained from Friday Night Lights. The arousal time course for Friday Night Lights was predicted from a model trained from Sherlock. This averaged predicted arousal time course (predicted arousal) significantly correlated with the averaged group-average arousal rating from a separate group of individuals (observed arousal). The gray bands indicate the standard deviation of predicted arousal across participants at each time point. Model-predicted arousal time courses also corresponded with the plot of each movie. In Sherlock, the most arousing moment predicted by the model occurred in the scene when the Sherlock Holmes was figuring out clues near a dead body, while the least arousing moment occurred during a long conversation between John Watson and his therapist; In Friday Night Lights, the most arousing moment predicted by the model occurred when a star player was severely injured in the ball game, while the least arousing moment occurred when the coach and a player having a conversation. Images in the figures are blurred for copyright reasons; in the experiment, movies were shown at high resolution. (TIFF) [file pcbi.1012994.s006.tiff]

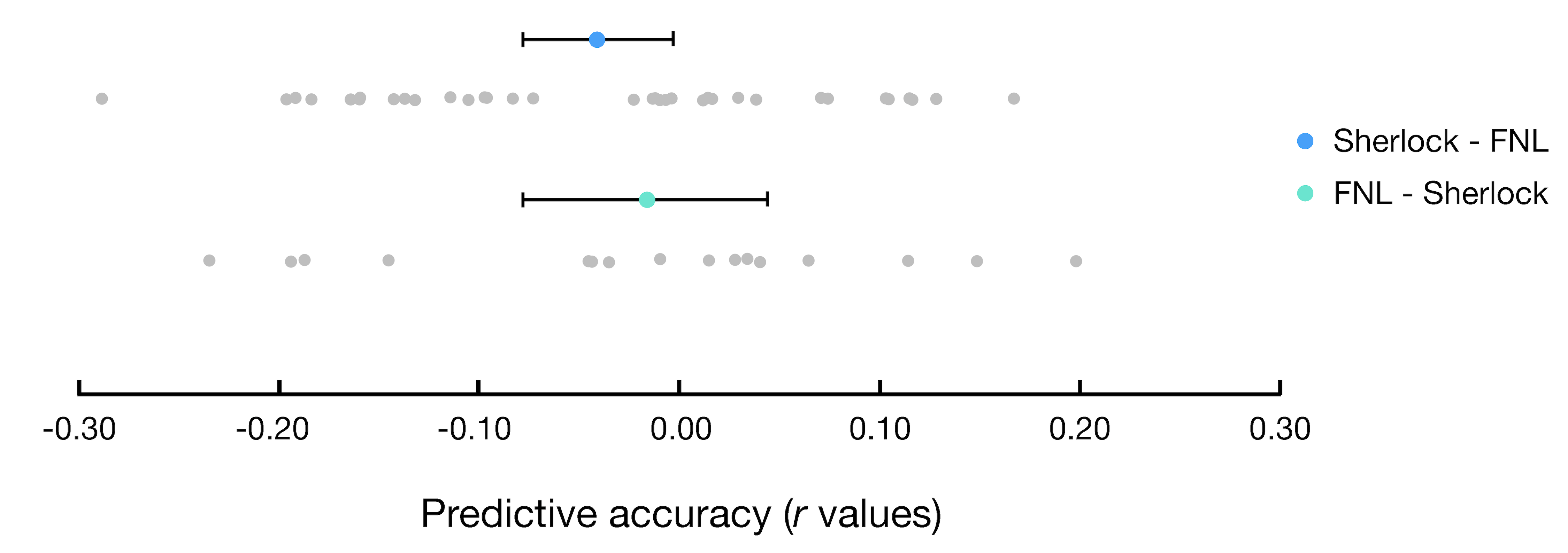

Supplement: S7 Fig — Equivalence tests were performed to assess whether the across-dataset predictive accuracies in predicting valence fell within a predefined range around zero, which would suggest that they were not only statistically non-significant but also practically insignificant. We defined an equivalence interval of [-.100,.100], with the bounds determined based on a small effect size of r = .100. Each datapoint in the box plot represents the predictive accuracy of each round of cross-validation. The black horizontal lines show the 95% percent CI of the mean r value. The equivalence test was significant for both across dataset accuracies (Sherlock - Friday Night Lights: p = .368, Friday Night Lights - Sherlock: p = .306), indicating that the observed predictive accuracies were statistically indistinguishable from zero within the defined bound. (TIFF) [file pcbi.1012994.s007.tiff]

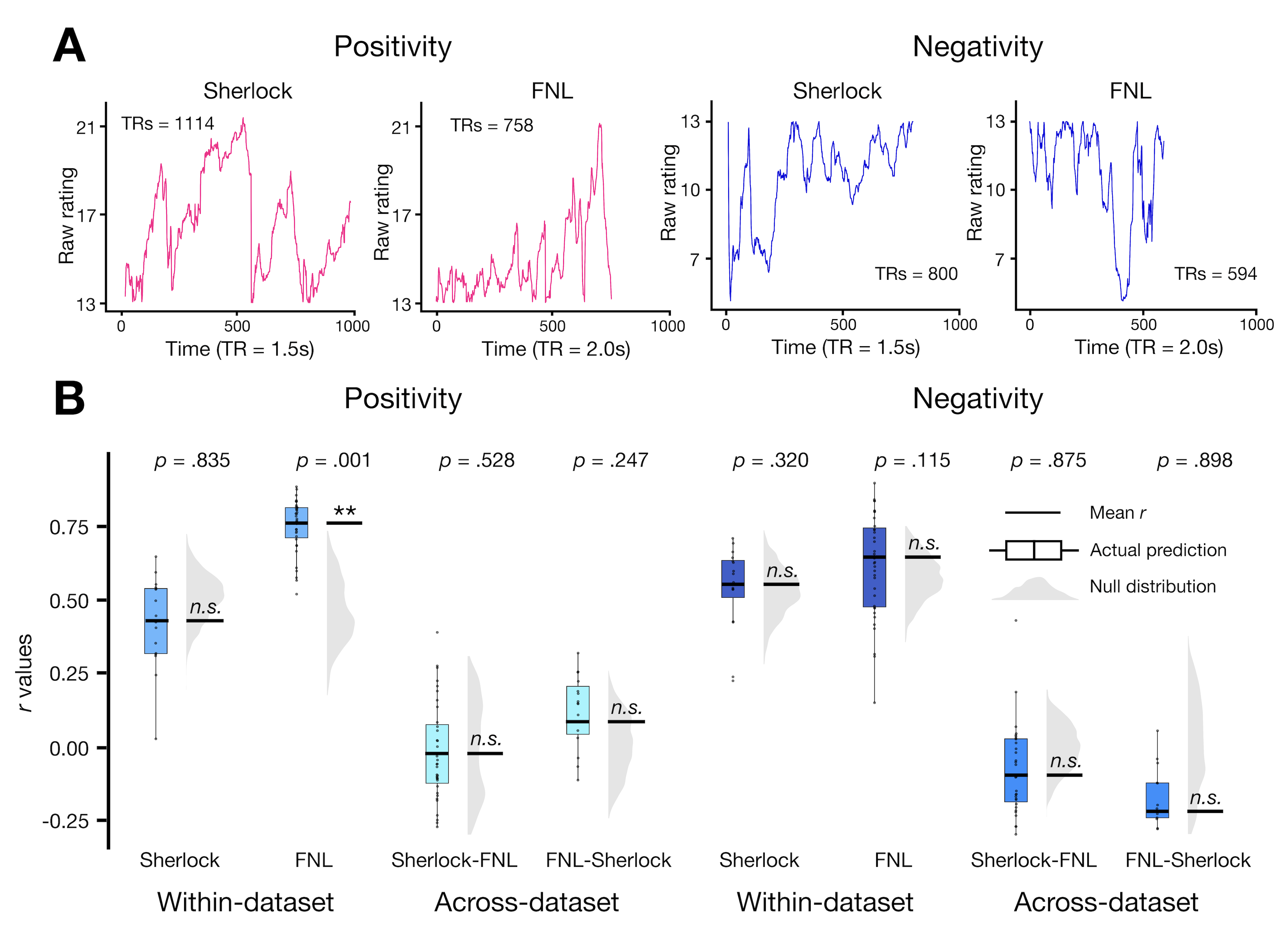

Supplement: S8 Fig — A Participants’ subjective affective experience of positivity (left) and negativity (right) fluctuates over time during naturalistic movie watching. B. Dynamic functional connectivity does not predict subjective feelings of positivity and negativity. CPM performance in predicting positivity and valence for within-dataset (the left panel) and between-dataset (the right panel). The y-axis represents the predictive accuracy, as measured by Pearson’s correlation between the model predicted time course and the observed group-average time course. Each datapoint in the box plot represents the predictive accuracy of each round of cross-validation. The black horizontal lines show the Fisher-z transformed mean r value. The gray half-violin plots show the null distribution of 1000 permutations, generated by phase-randomizing the observed group-average before training and testing the models. *: p < 0.05, **:p < 0.01, n.s.: p > 0.05, as assessed by comparing the empirical mean predictive accuracy against the null-distribution. (TIFF) [file pcbi.1012994.s008.tiff]
