# Supplementary material for: Dynamic brain connectivity predicts emotional arousal during naturalistic movie-watching
Source: PLoS Comput Biol. 2025 Apr 11;21(4):e1012994. doi: 10.1371/journal.pcbi.1012994 (PMC12058195; doi:10.1371/journal.pcbi.1012994)
Supplement: Attachment — Submitted filename: Ke_ResponseLetter.pdf [file pcbi.1012994.s009.pdf]

Revision Cover Letter

We are grateful for the thoughtful and detailed review provided by the three Reviewers. The comments have been invaluable as we revise our manuscript for submission to *PLOS Computational Biology.* Below is a summary of key revisions, followed by a point-by-point response to the Reviewers' comments:

1. **Clarifying the Contribution:** We have revised the manuscript to more accurately reflect the scope and contribution of our work: predicting emotional arousal during narrative viewing from whole-brain dynamic connectivity. We believe that developing a predictive model of emotional arousal that generalizes across datasets and rich, complex narratives is a significant advance over previous work and would be of interest to the affective science and computational cognitive neuroscience communities.

2. **Removal of Multivariate Activity Pattern-Based Analyses:** Reviewers 1 and 2 raised concerns about the multivariate pattern-based results, noting that they were inconsistent with prior literature and that the differences might be attributable to methodological factors. Given the extensive body of work on multivariate pattern-based models, we believe further exploration of these analyses would not meaningfully enhance the manuscript and could detract from our primary focus on the connectivity-based predictive models. Due to the lack of clarity in the interpretation of these results and to maintain a clear, focused narrative, we have removed the pattern-based analyses and concentrate exclusively on the connectivity-based findings.

3. **Highlighting the distinction between emotional arousal and other related constructs:** We will clarify that our work specifically focuses on emotional arousal (or "affective" arousal), as measured through self-report, which is distinct from other forms of arousal, such as "wakefulness" arousal (typically measured in humans using EEG) and "autonomic" arousal (typically measured using physiological measures like pupil dilation or skin conductance). While our current study does not test whether the model generalizes to these other forms of arousal, we recognize that this is an important and exciting direction for future research. We will emphasize this distinction in the revised manuscript and suggest it as a potential avenue for further investigation.

With our submission, we have attached a clean version of our manuscript, as well as a version with changes highlighted in red font.

Sincerely,

Jin Ke
Graduate Student
Department of Psychology
Yale University

Yuan Chang Leong, Ph.D.
Assistant Professor
Department of Psychology
University of Chicago

Reviewer #1

Comment 1.1:

1. The suggestion that "The relationship between whole-brain functional connectivity patterns and affective experience, however, remains largely unexplored" seems disconnected from the literature. There is a fairly large body of existing research using whole-brain patterns of fMRI activity and dynamic functional connectivity to predict measures of arousal (and related constructs, see references below). There are meaningful gaps in the literature that this manuscript fills (in its use of complex narrative stimuli and self-report as the outcome of prediction), but the degree of advance seems overstated.

a. Loos, E., Egli, T., Coynel, D., Fastenrath, M., Freytag, V., Papassotiropoulos, A., ... & Milnik, A. (2019). Predicting emotional arousal and emotional memory performance from an identical brain network. NeuroImage, 189, 459-467..

b. Han, J., Ji, X., Hu, X., Guo, L., & Liu, T. (2015). Arousal recognition using audio-visual features and FMRI-based brain response. IEEE Transactions on Affective Computing, 6(4), 337-347.

c. Tobia, M. J., Hayashi, K., Ballard, G., Gotlib, I. H., & Waugh, C. E. (2017). Dynamic functional connectivity and individual differences in emotions during social stress. Human brain mapping, 38(12), 6185-6205.

d. Raz, G., Touroutoglou, A., Wilson-Mendenhall, C., Gilam, G., Lin, T., Gonen, T., ... & Barrett, L. F. (2016). Functional connectivity dynamics during film viewing reveal common networks for different emotional experiences. Cognitive, Affective, & Behavioral Neuroscience, 16, 709-723.

e. Kim, J., Weber, C. E., Gao, C., Schulteis, S., Wedell, D. H., & Shinkareva, S. V. (2020). A study in affect: Predicting valence from fMRI data. Neuropsychologia, 143, 107473.

We thank the Reviewer for pointing this out. We have removed this sentence and revised the manuscript to more accurately reflect the current state of the literature:

> Prior research has identified several brain regions where activity correlates with valence and arousal, including the orbitofrontal cortex (OFC), amygdala, ventral striatum, and insula [23–29]. More recently, there is growing evidence that patterns of dynamic connectivity between brain regions may also contain information about affective experience [30–33]. For example, a recent study by Young and colleagues [33] presented participants with a stressful movie clip and showed that connectivity between the salience network was associated with fluctuations in heart rate, a measure of physiological arousal. However, this study focused on a single movie stimulus, leaving it unclear whether the same connectivity-arousal relationship would generalize to other stimuli with different situational contexts. Moreover, it remains uncertain whether the same networks would be engaged when probing “emotional arousal”, where individuals have subjective awareness of their level of arousal and can report them behaviorally [34]. (Introduction, p. 3)

We have also revised the introduction to highlight the three ways in which our manuscript advance prior work (paragraphs 4-7): (1) focusing on whole-brain functional connectivity dynamics, rather than connectivity between specific brain regions or networks, (2) examining continuous self-reported affective experiences using complex, narrative stimuli, and (3) assessing out-of-sample generalizability by testing our predictive models on narratives and participants that were not used to train the model.

Comment 1.2:
2. One difference from much of the existing literature is the combination of cinematic stimulus materials with a narrative component and self-report as the outcome measure. Many theories hold that the brain contains circuitry that evaluates the valence of more basic stimuli (e.g., tastes, odors, static visual content) that do not have the rich temporal structure of narratives. The present work operationalizes valence and arousal as self-reported experiences during film viewing, which varies slowly over time. Evaluations of valence or arousal presumably occur to highly varied stimuli at much faster timescales than those measured. Although the authors suggest that the present work provides a more nuanced test of valence and arousal, it is unclear whether the CPMs developed can detect arousal-related changes to more conventional, punctate stimuli (e.g., arousal affective images or sounds). This would appear to be a necessary property of a generalizable model of arousal.

We appreciate the Reviewer's observation regarding the potential limitations of using narrative stimuli, which evoke slow, continuous changes in emotional arousal. While we agree that generalizing arousal models to more punctate, momentary stimuli would be valuable, it is beyond the scope of the current study. This is in part because our modeling approach relies on dynamic functional connectivity patterns calculated using a sliding window (see Fig. S2 for the effects of different window sizes), and are not well-suited for short, punctate stimuli that are often only 2-4 seconds long.

The core goal of our work is to demonstrate that emotional arousal can be reliably predicted during dynamic, naturalistic stimuli, where emotional experiences unfold continuously over time. In using the term "generalizable," our intention was to emphasize that the model generalizes across different narratives, people, and datasets. It was not our intention to suggest that our model would necessarily generalize to momentary, punctate stimuli. That said, we recognize the importance of extending these models to such stimuli and consider it an important avenue for future research. We have revised the manuscript to make clear the scope of our study and to explicitly mention the limitations regarding the model's applicability to punctate stimuli. We also highlight this as a potential direction for future work.

> Our study focused on predicting emotional arousal during dynamic, naturalistic stimuli, where affective experiences vary continuously over time. While this approach is valuable in that it mirrors how emotions develop and evolve in response to unfolding events, it is unknown whether the model would generalize to brief, momentary stimuli such as affective images [26,32] or sounds [3,4,27] that are also known to modulate emotional arousal. Testing this generalization is beyond the scope of the current study, in part because our modeling approach relies on dynamic connectivity patterns calculated using a sliding window approach and is not well-suited for stimuli presented over very short durations (e.g., 2-4 seconds). However, we recognize this as an important avenue for future research. New methods for computing dynamic connectivity at finer temporal resolutions (e.g., [78]) could help extend the current modeling framework to capture emotional arousal in response to momentary stimuli. (Discussion, p. 16-17)

Comment 1.3:
3. The language used to describe "encoding" is inconsistent with its standard use in the neuroscience literature. Typically, a measure of brain activity (e.g., firing rates or BOLD responses) is thought to encode a variable if the neural measure can be predicted using said variable. An encoding model of valence or arousal would use a one-dimensional feature set (or perhaps some other low-dimensional basis set) to predict patterns of brain activation or coactivation. The present work develops multiple decoding models that attempt to predict valence, arousal, and engagement based on connectivity measures. The reported findings

suggest that correlations between BOLD signals measured during free-viewing contain generalizable information about arousal, but not valence. I do not think they can be used to make claims about how valence and arousal are encoded in the brain.

Our work does indeed employ decoding models rather than encoding models. To avoid any confusion in terminology, we have replaced "encoding" with either "decoding" or "predicting" wherever applicable throughout the manuscript, including changing the title of the manuscript to "*Predicting emotional arousal during naturalistic movie-watching from whole-brain dynamic connectivity across individuals and movie stimuli*."

Comment 1.4

4. The randomization-based null models show very positively biased distributions in the training dataset (correlations around .5), but they are centered around zero when tested out of sample. These effects should be reported more clearly in the main text of the manuscript, as they are suggestive of overfitting the single stimulus used in the training dataset

We thank the Reviewer for the suggestion to emphasize this point. We have revised the manuscript to elaborate on the positively-biased and zero-centered null distributions when testing within- and across-datasets respectively.

> Statistical significance was assessed by comparing model accuracy against a null distribution of 1000 permutations generated by training and testing the model on phase-randomized behavioral ratings. This null distribution provided a conservative test of model performance when training and testing within the same dataset, as it indicates a baseline measure of model performance when the model overfits to non-specific associations in a single stimulus. Indeed, the observed null distribution was positively skewed (centered around 0.5 rather than 0; Fig. 3A, left), suggesting that the null models learned associations between FC connections and arbitrary stimulus features that correlated with the phase-randomized behavioral time course in each iteration. (Results, p. 8)
>
> …
>
> To test model generalizability, we trained a CPM in one dataset on its arousal network to predict the group-average arousal time course and applied the trained model to the other dataset with a different group of participants watching a different movie. A null distribution was again generated by training and testing on phase-randomized behavioral ratings. However, in this case, the training and test data consists of different stimuli, thus any non-specific associations learned by the model from the training dataset would not generalize to the test dataset. As expected, the null distribution was centered around zero (Fig. 3A, right).
>
> The across-dataset predictions of arousal (Fig. 3A, right) were significantly above chance both for a model trained on Sherlock and tested on Friday Night Lights (mean $r = .270$, $p = .010$; RMSE = .977, $p = .009$), and a model trained on Friday Night Lights and tested on Sherlock (mean $r = .198$, $p = .001$; RMSE = .999, $p = .001$). These results indicate that our models generalized across movies and were not merely overfitting to a single movie. (Results, p. 8-9)

Comment 1.5

5. Comparisons are made between CPMs trained to predict self-reported engagement and arousal, as these two variables are correlated in the training dataset. The reported results show that a CPM trained to predict engagement performs modestly better at predicting self-reported engagement than an arousal model. Does a CPM trained to predict arousal perform better at

predicting arousal compared to engagement? A double dissociation would be required to conclude that the CPMs are picking up on two distinct sources of information (arousal and engagement networks).

We thank the Reviewer for this excellent suggestion. We have run the suggested analysis and found a double dissociation. We have revised the manuscript to include these results:

> However, model-predicted engagement was not significantly correlated with behavioral arousal ratings (mean r = .498, p = .173; MSE = .754, p = .163). Moreover, this correlation was significantly lower than that between model-predicted arousal and behavioral arousal ratings (r = .498 vs. .558; paired-t test, $t(15)$ = - 4.368, p < .001). Likewise, model-predicted arousal ratings were not significantly correlated with behavioral engagement ratings (mean r = .518, p = .172; MSE = .735, p = .167), and this correlation was significantly lower than that between model-predicted engagement ratings and behavioral engagement ratings (r = .518 vs. .572; paired-t test, $t(15)$ = - 4.115, p < .001). In other words, even though behavioral ratings of engagement and arousal were correlated, we found a double association such that model-predicted arousal was associated with behavioral arousal ratings, and model-predicted engagement was associated behavioral engagement ratings, but neither model accurately predicted the other measure. These results suggest that attentional engagement and arousal are related but non-synonymous constructs, with arousal possibly comprising an aspect of attentional engagement [50, 60]. (Results, p. 11)

Comment 1.6

6. The methods used for pattern-based predictive models seem outdated. Regularized regression (PCR, PLS, LASSO, etc.) rather than voxel-wise feature selection would be more comparable with the literature.

Our original intention was to use procedures that best matched those for training CPMs to facilitate comparison between the two approaches. However, in considering the reviews and the extensive body of work on pattern-based predictive models of affective experience, we have removed the pattern-based results from the manuscript as they are not central to our primary claims. Instead, our manuscript now focuses exclusively on predicting emotional arousal from dynamic functional connectivity patterns (see also our responses to Comments 1.8 and 2.1).

Comment 1.7

7. The statement that "These results provide a measure of the model's ability to predict arousal fluctuations of an average individual watching a movie that the model was not trained on, and suggest that our model could be used to generate moment-to-moment predictions of arousal during movie watching in new, independent datasets." is inconsistent with the analysis performed and overstates the findings. A group-level model was assessed on group-average behavioral measures. The results reflect performance on a group average, not a representative or average subject.

We thank the Reviewer for pointing this out and have revise the statement to accurately reflect this analysis:

> These results provide a measure of the model’s ability to predict the average arousal time course from a different group of participants watching a movie that the model was not trained on, and suggest that our model could be used to generate moment-to-moment predictions of arousal during movie watching in new, independent datasets. (Results, p. 13)

Comment 1.8

8. The statement that "These results suggest that while multivariate patterns capture arousal in specific contexts, connectivity patterns may tap into broader, stimulus-general neural mechanisms. In particular, the connectivity patterns may reflect the influence of global neuromodulatory systems, such as the norepinephrine system, which is known to regulate state-level changes across the brain and is crucial for modulating arousal in response to environmental demands" is not supported by the reported findings or sufficiently connected with the existing literature on valence and arousal (see references cited above). There are a number of studies that train pattern-based predictive models on different types of affective stimuli that generalize across tasks and stimulus types (e.g., Ceko et al. 2022 Nature Neuroscience, Kragel et al. 2023 Nature Human Behavior. It may be the case that pattern-based methods can be more sensitive to context, but there is evidence that when they are trained across multiple stimulus types, they can generalize. There are also studies reporting that pattern-based predictive models can generalize across stimulus types. For instance, see generalization tests of the Neurological Pain Signature developed by Wager's group, which some have argued is related to salience or nonspecific arousal.

We acknowledge that our interpretation of the pattern-based prediction results is inconsistent with the existing literature. As the reviewer correctly points out, there is significant evidence that pattern-based predictive models can generalize across various affective stimuli. We recognize there are several reasons why our pattern-based models did not demonstrate generalizability. As the reviewer suggests, one reason could be that the model was not trained on multiple stimulus types. Additionally, alternative methods, such as those mentioned by the reviewer in Comment 1.6, may be more sensitive in detecting generalizable patterns.

Considering the extensive prior work on pattern-based predictive models, we believe that further exploration of these analyses would not meaningfully enhance the manuscript and could detract from our core focus on the connectivity-based predictive models. Given the lack of clarity on these results, we have removed the pattern-based analyses from the manuscript and focus solely on the connectivity-based results. Additionally, we have added text that discusses our results in relation to these pattern-based results.

> Our connectivity-based predictive models complement prior work that predicts affective experience from multivariate activity patterns [29,70,73,74,78]. For example, Čeko and colleagues [74] demonstrated that negative affect could be predicted from distributed multivariate activity patterns, and that the predictive model generalized across negative affect induced by mechanical pain, thermal pain, aversive sounds and aversive images. More recently, work by Zhang and colleagues [78] predicted emotional arousal from whole-brain activity patterns, and showed that their predictive model also generalized across movies and individuals. Here, we show that connectivity patterns also contain information about emotional arousal. Specifically, our findings highlight that functional connections within and between multiple networks—including the ventral attention network, the frontoparietal network, the dorsal attention network, and the default mode network—were associated with continuous fluctuations in emotional arousal. This suggests that network interactions, in addition to regional activity patterns, may play a key role in tracking emotional arousal over time. (Discussion, p. 16).

Comment 1.9

9. The discussion covers many topics that are not directly supported by findings from the present work and is accordingly somewhat speculative. Whether the CPM trained to predict self-reported arousal also tracks variation in neuromodulatory, autonomic (e.g., pupil dilation,

skin conductance, or cardiorespiratory measures), or attentional measures that often correlate with subjective arousal is unknown.

We have revised the discussion to remove overly speculative claims that are not directly supported by the present findings. We have also added text in the discussion explicitly stating that it is unknown whether our model generalizes to autonomic measures, and suggesting this as an important avenue for future research.

> An additional consideration is the complexity and heterogeneity of arousal as a psychological construct [37,80]. In a recent review, Satpute and colleagues [37] deconstructed arousal into three varieties: wakefulness arousal, typically measured using EEG desynchronization; autonomic arousal, typically assessed via physiological measures such as pupil dilation or skin conductance; and (3) affective (i.e., emotional) arousal, typically measured through self-report. Overlapping and distinct neural mechanisms are thought to underlie the three varieties of arousal. Our study focuses specifically on emotional arousal, and our use of self-report is consistent with prior studies. However, how emotional arousal relates to wakefulness and physiological arousal during movie watching is unclear. Indeed, prior work suggests different varieties of arousal may diverge [78,81,82], and it is unknown whether our predictive model would generalize to the other two types of arousal. Our approach of testing whether predictive models trained on one dataset generalize to others paves the way for future studies to explore whether models trained on one form of arousal can predict another. This line of research could reveal overlapping neural representations across different varieties of arousal. (Discussion, p. 17)

Reviewer #2:

The aim of this study is to determine whether valence and arousal are encoded in generalizable neural representations represented within functional brain networks. To test their hypotheses, the authors collected continuous valence and arousal ratings from 120 participants when they watched movies outside of the scanner. These rating data were then used in the following analyses. They utilized connectome-based predictive modeling (CPM) to predict valence and arousal from whole-brain functional connectivity (FC) time series data. The results indicated that CPM showed significant predictions for arousal within both the original dataset and independent datasets, demonstrating its generalizability across three distinct fMRI datasets. However, CPM could not provide reliable prediction for valence. Additionally, the authors compared CPM with multivariate-based predictive modeling (MPM) and found that CPM had significantly higher predictive accuracy. This approach is novel and findings are interesting. However, I have several major and minor concerns about this study after reading it. Below are my concerns.

We thank the Reviewer for their careful read and thoughtful feedback on the manuscript. We are glad to hear that the Reviewer finds our findings novel and interesting. We have taken the Reviewer's comments to heart and revised the manuscript extensively. This includes removing the MPM analyses from the manuscript (see Comment 2.1) to focus on the connectivity-based predictive modeling results, which we see as the core advance of our work. We have also worked to address the Reviewer's major and minor concerns in detail and hope that the Reviewer will find our revisions satisfactory.

Comment 2.1

- Major concerns:

- My first major concern is that findings from this study were not consistent with findings from several well-powered published studies, in particularly that the MPM approach was unable to identify generalizable representations of valence. In Chang et al's study, (2015, PLOS Biology), the authors were able to take a MPM approach to establish a sensitive and specific neural

signature for negative-valenced affect. In addition, in a recent paper published in Science Advances from Kim et al. (2022), the authors were also able to establish a valence brain model with a MPM approach. Regarding the generalizable multivariate representations of arousal, a recent preprint from Zhang et al. showed that they were able to establish a predictive arousal brain signature by using naturalistic fMRI data with a MPM approach. These previous results demonstrated that brain models of valence and arousal were able to be established by taking a MPM approach, which was not in line with current findings. Importantly, not only static but also naturalistic data were collected in these studies, suggesting that it was not simply due to the nature of stimuli across datasets. Therefore, it is important to understand why the current study was unable to replicate findings from previous studies. The conclusion of MPM being not able to predict valence is risky.

We appreciate the reviewer's concerns regarding the multivariate pattern-based prediction analysis (MPM) results. Null results are notoriously difficult to interpret, and we believe there are many reasons why our MPM analyses could have failed. These include the lack of subjective ratings from the fMRI participants (see Comment 2.2), differences in prediction approach (see Comment 2.4), and the possibility of false negatives.

Given the lack of clarity on the MPM results and the extensive prior work that have successfully used MPMs to predict affective experience, we believe the MPM analyses reported in the previous version of the manuscript do not meaningfully add to our manuscript and have removed them accordingly. This allows us to focus on the connectome-based predictive modeling results, which we see as the core contribution of our study.

We have added text in the discussion to acknowledge the prior work using MPMs, as well discuss our results in relation to those studies:

> Our connectivity-based predictive models complement prior work that predicts affective experience from multivariate activity patterns [29,70,73,74,78]. For example, Čeko and colleagues [74] demonstrated that negative affect could be predicted from distributed multivariate activity patterns, and that the predictive model generalized across negative affect induced by mechanical pain, thermal pain, aversive sounds and aversive images. More recently, work by Zhang and colleagues [78] predicted emotional arousal from whole-brain activity patterns, and showed that their predictive model also generalized across movies and individuals. Here, we show that connectivity patterns also contain information about emotional arousal. Specifically, our findings highlight that functional connections within and between multiple networks—including the ventral attention network, the frontoparietal network, the dorsal attention network, and the default mode network—were associated with continuous fluctuations in emotional arousal. This suggests that network interactions, in addition to regional activity patterns, may play a key role in tracking emotional arousal over time. (Discussion, p. 16).

Comment 2.2

- Followed by the first concern, my second concern is related to the experimental design of the current study. The behavioral data corresponding to the four fMRI datasets were collected from separate, independent participants. Consequently, the analysis could only use each participant's brain data to predict an "average" behavioral response, rather than predicting the individual participant's own behavioral response. It would be beneficial to understand why the authors chose not to obtain both the fMRI data and behavioral data from the same participants during the experimental design phase. Does the difference in the experimental design lead to the inconsistency between findings from this study and findings from previous studies?

We used publicly available, open datasets for this study, which did not include affective ratings from the same participants as those who provided the fMRI data. However, we consider this to also be a strength, as it demonstrates that our model can be used to predict average emotional arousal in the growing number of open movie datasets, even in the absence of affective ratings. That said, we acknowledge the limitation of not having individual-level predictions and will revise the manuscript to include a discussion of the strengths and weaknesses of this design choice:

> The use of publicly available datasets, where fMRI and affective ratings were collected from separate participant groups, reflects both a strength and a limitation of our study. A strength is that connectivity-based arousal models trained on one group of participants generalized effectively to another, suggesting that neural representations of arousal are consistent not only across different stimuli but also among individuals. This design allows for training and testing in new samples, enhancing the applicability of our model to the growing number of open movie fMRI datasets. However, as we did not train and test on participant-specific arousal data, the model would not pick up on idiosyncratic neural representations of arousal. While our results demonstrate generalized representations of emotional arousal, this does not rule out the coexistence of individual-specific representations [87,88]. Thus, incorporating individual-specific data in future studies could further enhance prediction accuracy. (Discussion, p. 16)
>
> …
>
> Lastly, the neural representation of valence may be more person-specific when elicited by complex, meaningful contexts like narrative stimuli. In our study, we could not test this possibility, as the design did not include both subjective ratings and brain data from the same participants. Future work that collects both neural and behavioral data from the same participants may reveal whether person-specific patterns emerge during rich, contextualized emotional experiences. (Discussion, p. 18).

While the lack of subject-specific training data may have contributed to the inconsistency between our findings and those of previous studies, other factors, such as differences in the prediction algorithm, could also be responsible. Given the lack of clarity around the pattern-based results, we have removed those analyses and do not make any claims about MPM models in the revised manuscript.

<u>Comment 2.3</u>

- My third concern is related to the model-building process. During feature selection for model building, page 34 mentions, "In each round of cross-validation, we conducted feature selection, where functional connections (FC) that significantly correlated with affect time course in the training set of N - 1 participants were selected...". I would like to clarify whether the term "affect time course" refers to the empirical arousal time course? If so, does this imply that, during model training, the authors only selected features (x) that were significantly correlated with the ground truth (y)? Wouldn't this raise concerns about the model having prior knowledge of the ground truth? If not, this statement is somewhat confusing, and I would like to understand precisely what is meant by "affect time course" in this context.

“Affect time course” refers to the average behavioral time course (e.g., arousal or valence time course depending on the analysis).

We thank the reviewer for raising this important point. Following the Shen et al. (2017) protocol, best practices described in Scheinost et al. (2019), and prior dynamic CPM work (Song et al., 2021), we perform feature selection using data from N-1 subjects. This identifies functional connections whose dynamics track arousal dynamics in the training set. We then calculate the

strength of these connections in each time window using data from the held-out subject to predict their arousal time course. The arousal time course is therefore the same between the training and test subjects, inflating the correlation between predicted and observed arousal dynamics.

Importantly, however, we take two approaches to overcome this potential issue and show that predictive power is higher than what we would expect due to this inflation alone.

First, we conducted nonparametric permutation tests where we compare our result (Pearson's correlation between the actual and predicted behavioral time series) to a distribution created with 1,000 phase-randomized behavioral measures. This null model incorporates the same problems of synchronized FC time series and shared behavioral measures between train and test, which means we are equating the data dependence while testing the specificity of the real arousal time series. Our results indeed show that our prediction results were significantly higher than the null distribution. In other words, our within-dataset prediction success cannot simply be explained by the fact that the training and test arousal time courses are the same. If this were the case, our actual models would have performed no better than null models whose phase-randomized arousal time courses were also the same between training and test data.

Second, we emphasize that the across-data prediction has no data dependence whatsoever in cross-validation. The FC matrices of train and test were not collected from the same participant, nor in the same session or scanner, by different research groups and with different narrative stimuli. The behavioral measures of train and test were different because they were measured from different narrative stimuli. The findings that arousal prediction was successful even in this across-data prediction supports our argument that arousal dynamics can be predicted from FC.

We have revised the methods section to elaborate on our feature selection procedure and how our statistical procedures guard against over-fitting:

> Following prior CPM work [38,40,98], we perform feature selection using data from N-1 subjects. Specifically, in each round of cross-validation, we conducted feature selection, where functional connections (FC) that significantly correlated with affect time course (i.e., average behavioral valence or arousal ratings) in the training set of N - 1 participants were selected (one-sample Student's t test, $p < .01$).
>
> …
>
> As the affect time course is the same between training and test subjects viewing the same movie, the correlation between predicted and observed affect dynamics is inflated. To overcome this potential issue and assess that predictive power is higher than what we would expect due to this inflation alone, we conducted nonparametric permutation tests where we compared model performance against null distributions generated by training and testing the model on phase-randomized behavioral ratings (1000 permutations). These null distributions provide a baseline measure of model performance when training and testing on the same behavioral measure, and allow us to assess the specificity of our results to the affect time course of interest.
>
> …
>
> However, unlike the within-datasat FC prediction, training and test affect time course were different for across-dataset FC prediction because they were measured from different narrative stimuli. Above-chance prediction would thus indicate that the model generalizes across individuals and narratives, suggesting that the corresponding affect time course can be predicted from FC dynamics. (Methods, p. 21-22).

Shen, X., Finn, E., Scheinost, D., Rosenberg, M.D., Chun, M.M., Papademetris, X., Constable, T. Using connectome-based predictive modeling to predict individual behavior from brain connectivity. Nature Protocols 12, 506–518 (2017). https://doi.org/10.1038/nprot.2016.178

Scheinost, D., Noble, S., Horien, C., Greene, A. S., Lake, E. M., Salehi, M., ... & Constable, R. T. (2019). Ten simple rules for predictive modeling of individual differences in neuroimaging. NeuroImage, 193, 35-45.

Song, H., Finn, E. S., & Rosenberg, M. D. (2021). Neural signatures of attentional engagement during narratives and its consequences for event memory. Proceedings of the National Academy of Sciences, 118(33), e2021905118.

Comment 2.4

- Minor concerns:

- The authors conducted feature selection (24333 features) in their MVPA. I would like to know whether performing a whole-brain MVPA without feature selection might replicate the results of previous studies. In addition, when adapting the MPM approach, I would like to see results from SVR models with different kinds of kernels. The authors only used "rbf" now, but it might not be the most optimal option.

We thank the Reviewer for this suggestion. We were not able to predict valence from whole-brain MVPA without feature selection, nor when using a linear kernel. As described in Comment 2.1, we have removed the MVPA results from the manuscript.

Comment 2.5

- The concept behind Fig. 4B is commendable, but the visualization is overly cluttered, making it difficult to discern the intended message. It is recommended to either separate the positive and negative (red and blue) into distinct plots or to consider removing this figure.

We have separated positive and negative edges into distinct plots:

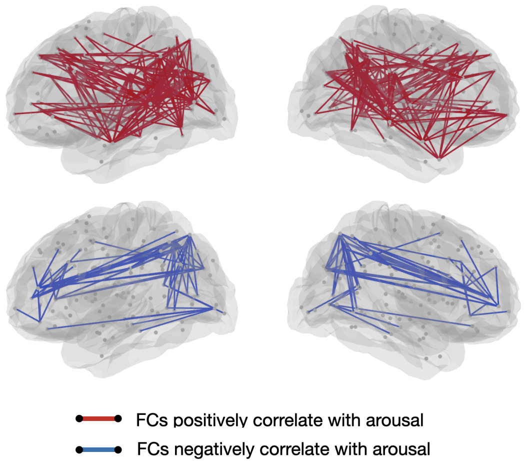

Figure 4B. Visualization of the overlap arousal network. Each node represents a brain region. The lines connecting two nodes show their functional connection (red: high-arousal network; blue: low-arousal network).

Comment 2.6

- In the last paragraph of p.32, it is mentioned that "Further fMRI analysis in the study involved the application of a tapered sliding window. In order to align the behavioral time courses to fMRI data time courses, we convolved the group-average time courses with the hemodynamic response function (HRF)...". I believe it would be beneficial to provide a detailed explanation of what is meant by "Further fMRI analysis," as this section is somewhat confusing. Specifically, I would like to know in which analysis the convolved behavioral time courses with the tapered sliding window were applied. Was this used in CPM, GLM or MPM?

By "further analysis," we meant "all subsequent analyses." We have revised the main text accordingly:

> All subsequent fMRI analyses in the study involved the application of a tapered sliding window. (*Methods*, p. 20).

Comment 2.7

- Page 20: The average engagement rating from this study was significantly correlated with the average arousal ratings (r =.778, p=.014). The r value seems not in line with its p value. Please make sure this result is correct.

Due to temporal autocorrelation in time series data, p-values computed from Pearson's r can result in inflated false positive rates. To control for this, we employed a stringent permutation procedure, where we compared the Pearson correlation coefficient against a null distribution generated by circularly shifting the engagement time course. This approach provides a more reliable estimate of the statistical significance by accounting for the inherent autocorrelation in the time series data. As such, the correlation coefficient may not match the p-value computed using a t-test.

Comment 2.8

- Mean r values (r = .498, .558) of predictions trained on engagement were not significant. However, the difference between these two r values (r = .498 vs. .558) was significant. The chance of showing such result was pretty low. Please make sure this result is correct.

We apologize for the confusion. To clarify, the mean *r* value of a model trained on arousal and tested on arousal was significantly above chance (r = 0.558, p = .024), while the mean *r* value of a model trained on engagement and tested on arousal was not (r = 0.498, p = 0.173). We then directly compare the two set of *r* values to show that a model trained on engagement and tested on arousal performed worse than one trained on arousal and tested on arousal (r = .498 vs. .558; paired-t test, t(15) = - 4.368, p < .001).

Reviewer #3:

This is a very well done study; I have little to suggest by way of further analyses, given the thoroughness of the present version. The study begins with a well articulated introduction that sets forth the questions. The overarching question of how arousal is represented in the brain, and whether it is a generalizable representation or varies across contexts and individuals is an important and long-standing question in affective science.

The approach is exemplary. All the best practices are used, in particular aiming for robust and reproducible results. Multiple approaches are tried, and held out datasets tested to ensure findings generalize broadly. A common confound is that arousal is associated with particular sensory features (e.g., loud and bright and fast moving stimuli etc.). This possibility is examined by regressing these lower-level features out. There are also examinations of multiple sliding window widths, of valence (as both bivalent and univalent), and comparison with MVPA as compared to CPM. Across all these many comparisons and controls, a CPM-based representation emerges as both reproducible and specific for arousal.

A next question is interpretability. The CPM results are queried as features and compared to the standard repertoire of resting-state networks to gain further insight into the neuroanatomical basis for the successful predictions.

We thank the Reviewer for their thoughtful and positive feedback. We appreciate the recognition of our efforts to address confounds and ensure generalizability, and we hope our work contributes meaningfully to understanding the neural representation of human affective experience.

Comment 3.1

One question left open regards the validity of the arousal ratings. The entire study is based upon explicit ratings of this concept. To be sure, it shows consensus across the rater, so they are doing something on which they can agree. But is it "arousal" or some other concept related to arousal? It would have been interesting to compare the present findings with other measures of arousal, such as for instance pupillary response while watching the movie (regressing out luminance effects, etc.) or EEG arousal if that were available. Given the prominent speculation in the paper's discussion that their network-based model may reflect NE effects, one might have expected some actual empirical link to this (e.g., PDR). As it stands, the sophistication of the fMRI analysis vastly outstrips the single measure of the psychological construct, which is based entirely on people's ratings of what they think they are feeling as "arousal". At a minimum, this issue should be addressed in a little more detail in the discussion.

We appreciate the reviewer's thoughtful feedback regarding the complexity of arousal as a construct. In a recent review, Satpute and colleagues (2019) deconstructed arousal into three varieties: (1) **wakefulness arousal**, typically measured using EEG de-synchronization; (2) **autonomic arousal**, often assessed via physiological measures such as pupil dilation or skin conductance; and (3) **affective (i.e., emotional) arousal,** which is the focus of our study and is most commonly measured through self-report. Some brain regions have been associated with all three types of arousal, while others are linked to only one or two.

Our use of self-report is consistent with how emotional arousal is usually measured in prior studies. As we lack measures of wakefulness and autonomic arousal in the current datasets, we are unable to directly test if our predictive model of emotional arousal would generalize to these other forms of arousal. While this is beyond the scope of the current study, we believe this to be an important and exciting avenue of future work.

In the revision, we have included additional discussion to clarify the distinctions between different types of arousal, emphasize that our study focuses on affective arousal, acknowledge that it is presently unclear whether the model will generalize to the other forms, and highlight this as an important area for future work.

Satpute, A. B., Kragel, P. A., Barrett, L. F., Wager, T. D., & Bianciardi, M. (2019). Deconstructing arousal into wakeful, autonomic and affective varieties. Neuroscience letters, 693, 19-28.

> An additional consideration is the complexity and heterogeneity of arousal as a psychological construct [37,79]. In a recent review, Satpute and colleagues [37] deconstructed arousal into three varieties: wakefulness arousal, typically measured using EEG desynchronization; autonomic arousal, typically assessed via physiological measures such as pupil dilation or skin conductance; and (3) affective (i.e., emotional) arousal, typically measured through self-report. Overlapping and distinct neural mechanisms are thought to underlie the three varieties of arousal. Our study focuses specifically on emotional arousal, and our use of self-report is consistent with prior studies. However, how emotional arousal relates to wakefulness and physiological arousal during movie watching is unclear. Indeed, prior work suggests different varieties of arousal may diverge [80–82], and it is unknown whether our predictive model would generalize to the other two types of arousal. Our approach of testing whether predictive models trained on one dataset generalize to others paves the way for future studies to explore whether models trained on one form of arousal can predict another. This line of research could reveal overlapping neural representations across different varieties of arousal. (Discussion, p. 17)
