# Supplementary material for: Dynamic brain connectivity predicts emotional arousal during naturalistic movie-watching
Source: PLoS Comput Biol. 2025 Apr 11;21(4):e1012994. doi: 10.1371/journal.pcbi.1012994 (PMC12058195; doi:10.1371/journal.pcbi.1012994)
Supplement: Attachment — Submitted filename: ResponseLetter.pdf [file pcbi.1012994.s010.pdf]

Reviewer 1

Review context: I am currently joining the review process for this paper after one round of review/revision at PLOS Comp Bio was already completed. However, previously I reviewed this manuscript and a revision thereof at a different journal. I have read the latest round of review, and the authors responses to it.

Overall evaluation: In my initial review of this paper (elsewhere) I stated "Generally, I found this to be a very strong paper methodologically, and one that thoroughly investigates and provides a clear answer to its research question." I think that this evaluation not only holds true, but that the paper has been considerably strengthened – both by addressing my and the other reviewers comments in that prior review process, and by the current review responses at PLOS Comp Bio. I believe it would make a valuable contribution to the literature in its current form.

We sincerely thank the reviewer for reviewing our manuscript a second time and for their thoughtful evaluation. We are grateful for the helpful feedback provided in the first round of review and for the kind words about our work.

Comment 1.1

Specific comments: Since my own comments on this paper were satisfactorily addressed in a prior review process, I will limit myself to commenting on one of the main issues which arose in which round of review: Two reviewers commented on perceived inconsistency between the MPM findings and other, prior decoding studies focused on valence and arousal. While I appreciate the difficulty of interpreting a null result, and the other reviewers' desire for an internally consistent literature, I would also caution against requiring new results to be consistent with old ones. If the past decades of the psych reform effort have taught us anything, it is that even "established" results frequently do not replicate for a wide variety of reasons. Requiring new findings to replicate old ones can reify what are actually noisy, inconsistent effects into artificially ironclad patterns. I'm not saying that that is necessarily happening here with respect to valence/arousal decoding MPMs. However, given the many differences between the present study and the past studies which have observed accurate MPMs in this space, I don't think that the inconsistency here is necessarily a negative indicator about the accuracy of the present findings. The authors have chosen to remove the MPMs, which I don't think was necessary, but does render this point is somewhat moot anyway.

We thank the Reviewer for the thoughtful comment and for recognizing the contribution of our MPM results. We are in complete agreement with the Reviewer on the importance of avoiding the reification of noisy, inconsistent effects. Considering the feedback from previous rounds of review, we ultimately concluded that including the MPM results might be misleading, as the methods we used for MPM diverged from how activity-based analyses are typically conducted. A more thorough investigation of different activity-based prediction approaches seemed necessary to draw definitive conclusions about the activity-based representation of valence, which we felt was beyond the scope of the current paper. For clarity and focus, we decided to remove the MPM results from the manuscript.

Reviewer 2

Ke and colleagues develop a predictive model mapping from dynamic functional connectivity to behavioral ratings of arousal during naturalistic movie-viewing. They show that this model generalizes across individual brains (leave-one-subject-out cross-validation) and, importantly, across quite different movies (different characters, events, themes, etc). I'm receiving a version of this manuscript from the journal that has already been reviewed and revised at least once; for example, it seems that the authors have removed a less-convincing non-connectivity-based predictive model based on the previous round of reviews. That seems fine to me; I read the current version of the paper prior to looking at the reviews/responses and it seemed coherent and complete on its own. Overall, the manuscript is well-written, the figures are excellent, the modeling and statistical analyses seem rigorous, and the authors include a number of useful control analyses to reinforce their conclusions. In sum, I think this manuscript is already in a very good place and essentially ready for publication. I provide a handful of relatively minor comments and suggestions below.

We thank the reviewer for the helpful suggestions and positive evaluation of our manuscript!

Comment 2.1

*My main comment is that I think the authors should be careful about how they set up and frame their work in terms of "individual-specific" neural signatures or "generalization across individuals". For example, in the Introduction (page 3), the authors write "One possibility is that these representations are specific to each situation and each individual" and ultimately contrast their findings with this possibility. But the authors are mapping individual brain activity onto a Y variable that's a *consensus* measure of arousal collected in a *separate sample* of subjects. This setup is not designed to find individual-specific neural signatures of arousal, because you don't have individual-specific behavioral ratings collected in the very same fMRI subjects "in the moment" while they watch the films. Put differently: in this setup, you either find a signature that generalizes across subjects or you don't; you would need behavioral ratings in the scanning subjects if you wanted to provide positive evidence for individual-specific neural signatures of arousal. I think the authors' current approach is perfectly fine and interesting enough in its own right; I would just encourage them to be careful in the writing not to inadvertently set up the reader to expect individual-specific results.*

We thank the Reviewer for this critique. We agree that our approach does not allow us to find positive evidence for individual-specific neural signatures of arousal. We have revised the manuscript to emphasize that our goal is to test for neural representations that generalize across individuals:

> The concept of valence and arousal as core components of affect has significantly influenced research and theories in emotion [18–20]. In particular, this perspective proposes an underlying similarity between excitement and anger due to both being high arousal experiences. Similarly, joy and gratitude are thought to be similar due to both being positively valenced. However, the extent to which these similarities extend to how the brain represents affective experiences remains an open question. Specifically, are there generalizable neural patterns associated with valence or arousal across various contexts? Moreover, given proposals that valence and arousal

> are processed by core neural systems [20,21], are these representations shared across individuals? (Introduction, p. 3)

Comment 2.2

In setting up the predictive modeling results (page 8), you state "In every round of cross-validation we selected FCs that significantly correlated with arousal in the training set as features." I suspect this line will tend to raise red flags for readers, whether justified or not. If I understand correctly, this feature selection strategy is fair (not double-dipping) because the edges are selected in the training set (N – 1 subjects); maybe just say this more explicitly? Or if there's a precedent in the field for doing feature selection in this way, maybe cite that as well? I suspect the implication that each training fold will use a slightly different set of features may bother people (although these feature sets should be highly correlated due to the similarity between training sets in leave-one-out cross-validation). Anyway, even just a single additional sentence here could preempt readers from becoming suspicious.

The Reviewer is correct in understanding that the edges were selected in the training set of N - 1 participants and tested on the held-out participant, thus the feature selection step does not involve the test data. Indeed, this procedure is consistent with prior work (Shen et al., 2017; Scheinost et al. 2019; Song et al., 2021). We have revised the manuscript to clarify this:

> In every round of cross-validation, we performed a feature selection step where we selected FCs that significantly correlated with the arousal time course in the training set of N - 1 subjects (one-sample Student's t test, $p < .01$), following procedures described in prior work [38,40,44,57]. The model was then tested on the held-out subject. Neither the model training nor the feature selection step involved the held-out subject, ensuring no data leakage. (Results, p. 7)

Shen, X., Finn, E., Scheinost, D., Rosenberg, M.D., Chun, M.M., Papademetris, X., Constable, T. Using connectome-based predictive modeling to predict individual behavior from brain connectivity. Nature Protocols 12, 506–518 (2017). https://doi.org/10.1038/nprot.2016.178

Scheinost, D., Noble, S., Horien, C., Greene, A. S., Lake, E. M., Salehi, M., ... & Constable, R. T. (2019). Ten simple rules for predictive modeling of individual differences in neuroimaging. NeuroImage, 193, 35-45.

Song, H., Finn, E. S., & Rosenberg, M. D. (2021). Neural signatures of attentional engagement during narratives and its consequences for event memory. Proceedings of the National Academy of Sciences, 118(33), e2021905118.

Comment 2.3

I'm still not sure I fully "get" why the within-dataset null distributions (e.g. Figure 3) are so high. In general, I think the statistical approach is sound and this positively shifted null distribution will only make the tests more conservative. But any extra effort to build an intuition for why this is the case up front would be appreciated.

Thank you for the suggestion. To clarify, for a given permutation iteration, the model was trained and tested on the same phase-randomized behavioral ratings, matching the procedure used in the within-dataset analysis. This means that the model can potentially learn arbitrary features correlated with the phase-randomized time course and use those for prediction during testing. By constructing the null distribution in this manner, we demonstrate that our CPM's ability to predict arousal goes above and beyond what would be expected if the model were simply learning non-specific features. Thus, this positively shifted null distribution is a conservative test of the specificity of predictive accuracy. We have revised the manuscript to make this reasoning clearer:

> Statistical significance was assessed by comparing model accuracy against a null distribution of 1000 permutations generated by training and testing the model on phase-randomized behavioral ratings. For a given permutation iteration, the model was trained and tested on the same phase-randomized time course. In other words, the model can potentially learn arbitrary features correlated with that iteration's phase-randomized time course and use those for prediction during testing. The resulting null distribution thus provides a conservative test of model performance when training and testing within the same dataset, as it reflects the baseline performance expected if the model learned arbitrary features associated with a stimulus. Indeed, the observed null distribution was positively skewed (centered around 0.5 rather than 0; Fig. 3A, left), suggesting that the null models learned associations between FC connections and arbitrary stimulus features that correlated with the phase-randomized behavioral time course in each iteration. (*Results*, p. 7)

<u>Comment 2.4</u>
I'm curious whether the authors have tried using intersubject functional connectivity (ISFC; Simony et al., 2016) rather than within-subject functional connectivity to drive their predictive model. ISFC would presumably isolate the stimulus-driven component of connectivity (maybe a good thing?), but might effectively filter out arousal signals that are not very precisely synchronized to the stimulus (maybe a bad thing?), and I suspect would ultimately reduce the generalizability of the model across different stimuli. Anyway, I understand this would complicate things and I don't think it's necessary for the current manuscript—just wondering if the authors have any intuitions or anecdotal evidence on how ISFC would affect their model.

We thank the Reviewer for this excellent question. We did not attempt ISFC as we aim to eventually extend our predictive modeling approach to setups without a shared stimulus (e.g., physiological arousal during resting state) or where interpretations are subjective (e.g., individual-specific ratings), which are incompatible with ISFC.

Nevertheless, our intuition is that ISFC, by leveraging between-subject similarity in functional connectivity, effectively performs an additional feature selection step by retaining only FC patterns that are consistent across participants and thus more likely to be associated with the stimulus. This could, in theory, improve prediction accuracy within a dataset. However, as the Reviewer notes, it is unclear how well such a model generalizes to new stimuli.

Comment 2.5

In Figure 2, it looks like the error bands around the mean arousal/valence ratings are fairly consistent across time. I'm wondering: have the authors looked at time points of the film where variance in ratings is low versus high? Again, I don't think this is necessary for the current manuscript—just curious.

We thank the Reviewer for this great question. We did not compare model predictive accuracy between moments of low and high rating variance, as the current study design is not well-suited for such analyses. As mentioned by the reviewer, there is limited variability in the variance around the behavioral ratings, likely due to the stimuli having unambiguous interpretations. A stimulus, or an experimental design, designed to evoke in different participants similar interpretations in some moments and different interpretations in others would be better suited for the analysis suggested by the Reviewer.

Comment 2.6

I appreciate the effort the authors have put into making their code and models publicly available—that's great! (Looking forward to seeing the behavioral rating data released publicly as well.)

We thank the Reviewer for acknowledging our efforts! We have made the valence and arousal behavioral rating data in all 4 movies (i.e., *Sherlock*, *Friday Night Lights*, *North by Northwest*, and *Merlin*) openly available to the public:
https://github.com/jinke828/AffectPrediction/tree/main/beh_ratings

Comment 2.7

Page 4: you say "30 in each condition"—at first I was confused about what "conditions" you were referring to, but now I understand you mean 2 movies (Sherlock, FNL) x 2 ratings (arousal, valence); you might want to articulate conditions sooner or adjust the wording

For clarity, we have revised the introduction to avoid referring to "conditions" before defining the term in the first paragraph of the Results section:

> We utilized two publicly available fMRI datasets of participants watching TV episodes (N = 16 [56] and N = 35 [57]; Fig. 1), and collected continuous valence and arousal ratings of the two episodes from a separate group of participants (N = 120, 30 for each pairing of affective rating and movie–e.g., arousal ratings for Sherlock) (Introduction, p. 4)
>
> We will refer to the pairing of an affective dimension and a movie as an "experimental condition" (e.g., Sherlock-valence). Thus, we have four experimental conditions with a sample size of 30 participants each. (Results, p. 5)

<u>Comment 2.8</u>

Figure 3 caption: "for within-dataset (the left panel) and between-dataset (the right panel)" feels like it needs another noun at the end (e.g. "cross-validation") or just take out the "for"

We have removed "for" in this sentence. The caption now reads:

> **Fig. 3. Dynamic functional connectivity predicts fluctuations in arousal but not valence.** CPM performance in predicting arousal (A) and valence (B) within-dataset (the left panel) and between-dataset (the right panel) (p. 7).

References:

Simony, E., Honey, C. J., Chen, J., Lositsky, O., Yeshurun, Y., Wiesel, A., & Hasson, U. (2016). Dynamic reconfiguration of the default mode network during narrative comprehension. Nature communications, 7(1), 12141.

Signed: Samuel A. Nastase

Reviewer 3

Comment 3.1

Ke and colleagues presented models for predicting subjective arousal ratings of a movie based on functional connectivity patterns from fMRI data. Subjective arousal ratings and fMRI data were collected from two separate groups of participants. The models were tested on 'unseen' datasets. The work is interesting and, in my view, makes a valuable contribution to the field.

We thank the Reviewer for their helpful feedback and kind words on our manuscript!

Comment 3.2

Key points

The paper presents an impressive computational approach to affective representation, and I particularly appreciate the generalization across different datasets. However, the insights regarding the representation of arousal and its connection to theory remain unclear. I would like to see a more in-depth discussion of the theoretical implications of these findings, as well as a closer examination of the information conveyed by the connectivity patterns.

Thank you for this suggestion. We agree that a more in depth discussion of the theoretical contributions of our results would enhance the manuscript. First, in identifying a neural representation of emotional arousal that generalizes across datasets, we show that the brain represents arousal states similarly across different contexts. This finding potentially provides a neural basis for theories of emotion proposing that arousal states are inherently confusable and susceptible to contextual misattribution. The presence of a generalizable neural representation of arousal suggests it may serve as a core substrate upon which emotional experiences are constructed and interpreted.

Second, our findings indicate that information about arousal states is not restricted to activity or connectivity within individual brain regions but is distributed across connections between multiple large-scale functional networks. One implication of this finding is that arousal may be encoded in the dynamic interactions between brain systems aligning with network-based models of affect and cognition. We have revised the manuscript to emphasize on these core contributions and added discussion on the specific patterns of connectivity:

> In identifying a neural representation of emotional arousal that generalizes across datasets, we show that the brain represents arousal states similarly across different contexts. This finding potentially provides a neural basis for theories of emotion proposing that arousal states are inherently confusable and susceptible to contextual misattribution. In particular, the two-factor theory of emotion proposes that emotional experiences arise from the combination of physiological arousal and the cognitive interpretation of that arousal [77]. To interpret arousal, individuals typically rely on contextual cues in the immediate environment. In cases where the source of arousal is ambiguous, people may mistakenly attribute their arousal to an incorrect source, a phenomenon known as the misattribution of arousal [78–81]. For example, a person who arrives at a job interview after climbing up a flight of stairs might mistakenly attribute the arousal from the physical activity to nervousness about the interview, which can in turn affect their performance on the interview. Our results suggest that such confusions may occur because the

brain represents arousal states similarly across different contexts. This shared representation of arousal could serve as a fundamental substrate upon which emotional experience is constructed and interpreted.

Our connectivity-based predictive models complement prior work that predicts affective experience from multivariate activity patterns [27,69,72,73,87]. For example, Čeko and colleagues [73] demonstrated that negative affect could be predicted from distributed multivariate activity patterns, and that the predictive model generalized across negative affect induced by mechanical pain, thermal pain, aversive sounds and aversive images. More recently, work by Zhang and colleagues [87] predicted emotional arousal from whole-brain activity patterns, and showed that their predictive model also generalized across movies and individuals. Here, we extend these findings by demonstrating that emotional arousal is not only reflected in regional activity patterns but also in large-scale connectivity between brain networks. Specifically, our findings highlight that functional connections within and between multiple networks—including the ventral attention network, the frontoparietal network, the dorsal attention network, and the default mode network—were associated with continuous fluctuations in emotional arousal. This suggests that network interactions, in addition to regional activity patterns, plays a role in tracking emotional arousal over time, which implies that arousal may be encoded in the dynamic interactions between brain systems, aligning with network-based models of affect and cognition [45]. In this view, affective states emerge from the coordinated activity of multiple brain networks rather than being localized to discrete brain regions.

The positive associations between arousal and connections linking the DMN and FPN, as well as the DAN and VAN, indicate that arousal may be facilitated by interactions between systems involved in internally directed cognition [88], executive control [89], and attentional orienting [90]. These findings are consistent with theories proposing that arousal enhances neural flexibility, allowing for efficient coordination between higher-order cognitive systems to regulate emotional and attentional states [91–93]. Conversely, the negative association between arousal and DAN-VIS connectivity suggests that heightened arousal may relate to reduced coupling between attentional control and early sensory processing. This could reflect a shift in neural resources away from lower-level visual processing and toward internally guided or top-down attentional mechanisms. Future work will be needed to tease apart the functional contributions of these network interactions and determine how they differentially support arousal-related changes in cognition. (*Discussion*, p. 14-15)

Comment 3.3

The two datasets used in the paper are unbalanced (statistical significance aside). For the Sherlock dataset, valence ratings are more consistent across raters compared to arousal ratings, but the fMRI data is based on a smaller number of participants. In contrast, the Friday Night dataset shows more consistent arousal ratings than valence ratings, and has a larger fMRI sample. Could the absence of valence results be due to this imbalance? This issue could be explored using the supplementary datasets, but unfortunately, the equivalent information is not available for the two additional datasets provided in the supplementary materials. Did the authors attempt to predict valence for these datasets as well? Are the results 'symmetric' across data sets (switching training and testing)?

We appreciate the Reviewer's thoughtful question. We note that the consistency of the valence ratings in *FNL* (mean r = 0.653) is higher than that of the arousal ratings in *Sherlock* (mean r = 0.502), yet, a model trained on the *Sherlock* arousal ratings generalized to *FNL* and vice versa. Thus, we think that the non-significant valence predictions are unlikely to be due to the *FNL* ratings being insufficiently consistent.

In our manuscript, we show that a valence model that was trained on the combined *FNL* and *Sherlock* data failed to predict valence in the *Merlin* and *North by Northwest*. (see section *Connectome-based models of valence failed to generalize even with more training data*). We adopted the Reviewer's suggestion and ran an analysis where we switched the training and testing datasets. Specifically, we trained a valence model on the combined *Merlin* and *North by Northwest* data and tested the model on *Sherlock* and *FNL*. This model, too, failed to predict valence in the test data (*Sherlock*: r = 0.076, p = 0.102; *FNL*: r = 0.034, p = 0.206).

The consistency of valence ratings was lower in *Merlin* (mean r = 0.576; N = 18) and *North by Northwest* (mean r = 0.394; N = 32) than *FNL*, thus it is not possible for us to rule out the possibility that the inability to predict valence was due to the greater idiosyncrasy of valence ratings. We have revised the discussion to highlight this possibility:

> In contrast to emotional arousal, we were not able to predict emotional valence from whole-brain dynamic functional connectivity. Null results are inherently difficult to interpret, but we offer several possible speculations. One reason could be that the experience of valence is idiosyncratic during movie-watching, in which case the group-average ratings from a different sample of individuals may not be representative of the experience of the individual participant. Thus, even if the model were successful at predicting the subjective experience of valence in a participant, the predicted output may not match the group-average rating time course of a different sample. Future work that collects subjective ratings and brain data from the same participants will be necessary to test this possibility. (Discussion, p. 16)

Comment 3.4

Other points

The authors state: "Such stimuli may not capture the diversity and complexity of real-world emotional experiences, raising questions about whether the neural representations of valence and arousal identified under these controlled conditions would generalize across varied and dynamic real-life situations, and across different individuals encountering these contexts. … allowing us to capture moment-to-moment affective responses across a variety of situations." However, the current study does not address this limitation either. Watching a movie is just one example of 'varied and dynamic real-life situations' and does not fully capture the complexity of such contexts.

We thank the Reviewer for highlighting this point. We agree that watching a movie does not fully capture the complexity of real-world emotional experiences. Our intention was to contrast the use of movies, which are rich and temporally extended stimuli with dynamic narrative arcs, against more commonly used decontextualized stimuli, such as isolated images or words. We argue that movies provide a step toward capturing more naturalistic and ecologically valid

emotional experiences, but acknowledge that they remain an artificial context and do not encompass the full range of real-life emotional situations. We have revised the manuscript to convey this more clearly:

> Movies, by contrast, provide extended, context-rich narratives that elicit dynamic and temporally continuous emotional responses [45,56,57]. While watching a movie is a passive, second-person experience and does not encompass the full range of real-life emotional contexts, they can serve as a valuable step toward understanding affective experiences in more ecologically valid contexts, where situations are complex, dynamic, and varied. Here, we examined the neural activity associated with continuous self-reported affective experiences during full-length TV episodes, allowing us to capture moment-to-moment affective responses across a variety of situations. (Introduction, p. 4)

Comment 3.5

Figure 4C: The term 'actual arousal' is misleading; in the figure it refers to the averaged subjective measure derived from a group of individuals distinct from those who provided the fMRI data. Please use a more descriptive label instead. Same applies to Supplementary figures.

We have replaced "actual arousal" with "observed arousal" in Fig. 4, Supplementary Fig. 5 and Supplementary Fig. 6. We have also revised the figure captions to explicitly state what the term refers to. For example,

> We averaged the model predictions across participants watching the same movie and computed the correlation between the group-average model predictions (predicted arousal) and group-average arousal ratings from a separate group of individuals (observed arousal). (Results, Fig. 4 caption, p. 10)

Comment 3.6

Discussion: "In line with this possibility, prior studies have successfully predicted the valence of stimuli, such as images, tastes, and words, from activity patterns [29,30,73,74,89]." Valence has also been predicted for movies (Kim et al., 2020).

We have revised the manuscript to incorporate this work:

> In line with this possibility, prior studies have successfully predicted the valence of stimuli, such as images, tastes, words, and movies, from activity patterns [27,28,72,73,94,99]. (Discussion, p. 16)

Comment 3.7

Infographics for rating and fMRI data in Figure 1 are not intuitive. How N=120 participants are allocated to conditions is unclear from the figure.

We have revised Figure 1 and the corresponding caption to clarify that there were 30 participants in each condition:

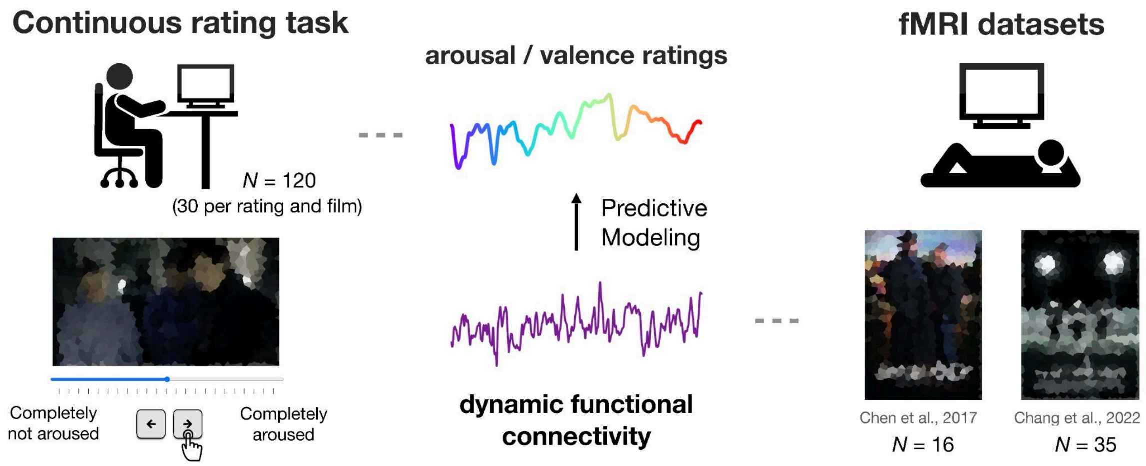

Figure. 1 Study schematic procedure. We utilized two publicly available fMRI datasets of participants watching Sherlock (N = 16) and Friday Night Lights (N = 35). Additionally, we collected behavioral ratings of the two episodes from a separate group of participants. Each participant is presented with one movie clip and is instructed to continuously rate how the clip is making them feel in terms of either valence (i.e., positive to negative) or arousal (i.e., not aroused to aroused). The 120 participants in the rating study were equally divided between conditions. Thus, there were 30 participants per pairing of an affective dimension (valence or arousal) and movie (*Sherlock* or *Friday Night Lights*). Images in the figures are blurred for copyright reasons; in the experiment, movies were shown at high resolution. We built within and across dataset CPMs to predict moment-to-moment affect ratings from dynamic whole-brain functional connectivity patterns.

Comment 3.8

The paper would benefit from careful editing.

- The abstract does not accurately reflect the content of the paper. It mentions that predictive models of both valence and arousal were built, but the text later creates confusion by focusing solely on the arousal model in the analysis. I believe this oversight occurred during the initial revision.

We thank the Reviewer for their careful read and feedback. While there is indeed a stronger emphasis on the arousal analyses in our manuscript, we do test and report predictive models of valence in the manuscript as well (see Fig. 3B, S7, S8, and the sections "*Dynamic functional connectivity does not predict moment-to-moment valence*" and "*Connectome-based models of valence failed to generalize even with more training data*" in the Results). Even though the valence model ultimately had chance-level performance, we felt that it was important to include valence results in the abstract to provide a complete account of the analyses performed.

Comment 3.9

- It is not clear at this point in the introduction what 'condition' is in reference to: "and collected continuous valence and arousal ratings of the two episodes from a separate group of participants (N = 120, 30 in each condition)."

Thank you for pointing this out. We have revised the introduction to avoid referring to "conditions" until we define the term in the first paragraph of the Results:

> We utilized two publicly available fMRI datasets of participants watching TV episodes (N = 16 [56] and N = 35 [57]; Fig. 1), and collected continuous valence and arousal ratings of the two episodes from a separate group of participants (N = 120, 30 for each pairing of affective rating and movie–e.g., arousal ratings for Sherlock) (*Introduction*, p. 4)
>
> We will refer to the pairing of an affective dimension and a movie as an "experimental condition" (e.g., Sherlock-valence). Thus, we have four experimental conditions with a sample size of 30 participants each. (*Results*, p. 5)

Comment 3.10

- Figure 1: How N=120 participants are allocated to conditions is unclear from the figure (is it 30 per rating per film?)

We have revised Figure 1 and the corresponding caption to clarify that there were 30 participants in each condition (please see our response to Comment 3.7).

Comment 3.11

'… correlations was significantly positive' is awkward

We have revised the sentence to read:

> The average intersubject correlation was significantly positive across all conditions (Results, p. 6)

Comment 3.12

- Figure 4C: what does the gray color represent?

The gray error bands represent the standard deviation of predicted arousal across participants at each time point. We have revised the Figure. 4 captions to make this clear:

> **C**. Connectome-based model trained on the overlap arousal network generalized to two more fMRI datasets, *Merlin* and *North by Northwest*. The left figure indicates how well the model could predict the group averaged experiences of arousal. We averaged the model predictions across participants watching the same movie and computed the correlation between the group-average model predictions (predicted arousal) and group-average arousal ratings from a separate group of individuals (observed arousal). The gray bands indicate the standard deviation of predicted arousal across participants at each time point. The right figure shows the predictive accuracy of the across-dataset prediction. The model was trained on a minimal set of FCs that predicted arousal in both *Sherlock* and *Friday Night Lights*, and tested separately on *Merlin* and *North by Northwest*. $^{**}p < .01$, as assessed by comparing the empirical mean predictive accuracy against the null distribution. (Results, Fig. 4 captions, p. 10)
